# The Lactic Acid Bacterium *Pediococcus acidilactici* Suppresses Autoimmune Encephalomyelitis by Inducing IL-10-Producing Regulatory T Cells

**DOI:** 10.1371/journal.pone.0027644

**Published:** 2011-11-15

**Authors:** Kazushiro Takata, Makoto Kinoshita, Tatsusada Okuno, Masayuki Moriya, Tohru Kohda, Josephe A. Honorat, Tomoyuki Sugimoto, Atsushi Kumanogoh, Hisako Kayama, Kiyoshi Takeda, Saburo Sakoda, Yuji Nakatsuji

**Affiliations:** 1 Department of Neurology, Osaka University Graduate School of Medicine, Suita, Osaka, Japan; 2 Department of Bio-medical statistics, Osaka University Graduate School of Medicine, Suita, Osaka, Japan; 3 World Premier International Immunology Frontier Research Center, Osaka University, Osaka, Japan; 4 Laboratory of Immune Regulation, Department of Microbiology and Immunology, Graduate School of Medicine, Osaka University, Suita, Osaka, Japan; 5 Department of Neurology, National Hospital Organization Toneyama National Hospital, Toyonaka, Osaka, Japan; Wayne State University, United States of America

## Abstract

**Background:**

Certain intestinal microflora are thought to regulate the systemic immune response. Lactic acid bacteria are one of the most studied bacteria in terms of their beneficial effects on health and autoimmune diseases; one of which is Multiple sclerosis (MS) which affects the central nervous system. We investigated whether the lactic acid bacterium *Pediococcus acidilactici*, which comprises human commensal bacteria, has beneficial effects on experimental autoimmune encephalomyelitis (EAE), an animal model of MS.

**Methodology/Principal Findings:**

*P. acidilactici* R037 was orally administered to EAE mice to investigate the effects of R037. R037 treatment suppressed clinical EAE severity as prophylaxis and therapy. The antigen-specific production of inflammatory cytokines was inhibited in R037-treated mice. A significant increase in the number of CD4^+^ Interleukin (IL)-10-producing cells was observed in the mesenteric lymph nodes (MLNs) and spleens isolated from R037-treated naive mice, while no increase was observed in the number of these cells in the lamina propria. Because only a slight increase in the CD4^+^Foxp3^+^ cells was observed in MLNs, R037 may primarily induce Foxp3^−^ IL10-producing T regulatory type 1 (Tr1) cells in MLNs, which contribute to the beneficial effect of R037 on EAE.

**Conclusions/Significance:**

An orally administered single strain of *P. acidilactici* R037 ameliorates EAE by inducing IL10-producing Tr1 cells. Our findings indicate the therapeutic potential of the oral administration of R037 for treating multiple sclerosis.

## Introduction

Multiple sclerosis (MS) is an inflammatory demyelinating autoimmune disease of the central nervous system (CNS). The number of patients with MS has been increasing, and MS is a leading cause of neurological disabilities in young adults [1]. Although the pathogenesis of MS is poorly understood, various mechanisms have been suggested by experiments utilizing experimental autoimmune encephalomyelitis (EAE), an animal model of MS [2]. A disturbed balance between effector and regulatory T cells has been thought to underlie the pathogenesis of the autoimmune inflammation of CNS [3]–[5]. Interleukin (IL)-17-producing T helper cells (Th17) and interferon-γ (IFN-γ)-producing T helper cells (Th1) play important roles in the induction and propagation of EAE/MS [3]. However, IL-10-producing regulatory T cells suppress these effector T cells. The critical role of IL-10 in the pathogenesis of EAE is supported by the facts that IL-10-defficient mice exhibit severe EAE, while IL-10-transgenic mice are EAE resistant [6].

Accumulating evidence suggests that intestinal microbiota affect systemic immune regulation. Because antigens associated with luminal bacteria were picked up and delivered to intestinal dendritic cells (DCs) to initiate T cell response, these microbiota are relevant to the pathogenesis of various autoimmune diseases [7]. In patients with inflammatory bowel disease, the subset of intestinal microbiota is remarkably imbalanced [8]. Th17 cells largely exist in the intestinal lamina propria (LP) in a steady state, and microbiota play an important role in the induction of these cells [9]. Segmented filamentous bacteria induce intestinal Th17 cells and influence various autoimmune disease models including an inflammatory arthritis and EAE [10]–[12]. IL-10-producing CD4^+^Foxp3^+^ T cells and IL-10-producing CD4^+^Foxp3^−^ T regulatory type 1(Tr1) cells comprise the major regulatory populations in the small intestine, and these cells control Th17 cells in IL-10 dependent manner [13]. Recent studies have shown that oral inoculation of indigenous Clostridium suppresses dextran sodium sulfate-mediated colitis, a model of human inflammatory bowel disease and increases antigen-specific IL-10 production from splenocytes by inducing colonic regulatory T cells. This observation implies that the alteration of intestinal microflora and modulation of intestinal immunity could be a novel approach to the treatment of autoimmune diseases [14].

Some lactic acid bacteria exist in human commensal microbiota, and they have been used in food and drinks for hundreds of years; microbial food ingredients beneficially affect the health of the host [15]. Many studies have suggested beneficial effects of lactic acid bacteria on various immune-mediated diseases. For example, *Lactobacillus plantarum* is effective in the colitis model, *Bifidobacterium lactis* and *L. acidophilus* in the food allergy model, and *L. casei* in the arthritis model [16]–[18]. However, only a little information regarding the effects of lactic acid bacteria on EAE/MS exists [19]. In the present study, we demonstrate that oral administration of *P. acidilactici* induces IL-10-producing regulatory T cells and suppresses EAE. *P. acidilactici* has been used as a starter culture for fermented sausages and is one of the human commensal bacteria [20], [21]. These data suggest that *P. acidilactici* can be a novel target for the treatment of human MS.

## Results

### Administration of the lactic acid bacterium *P. acidilactici* ameliorates EAE

**Figure 1:**
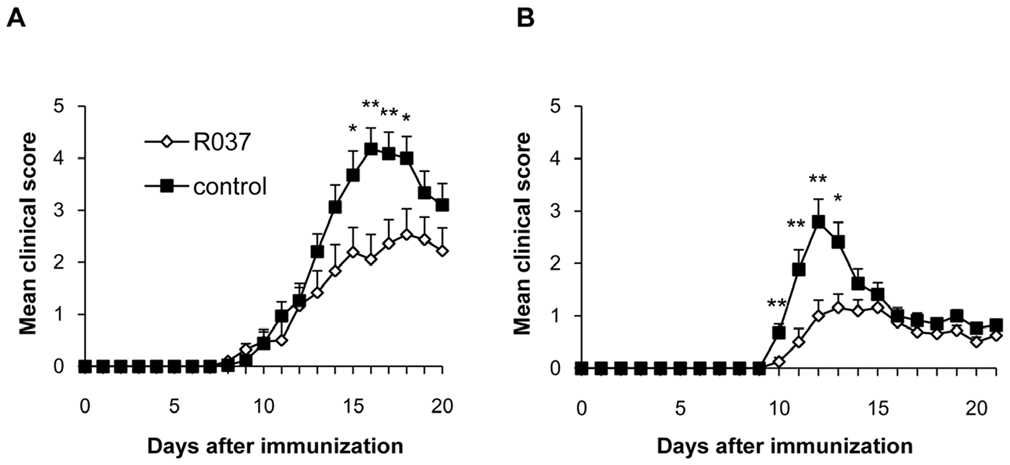
*Pediococcus acidilactici* ameliorates EAE. (A) R037 (4 mg/day; n = 18) or PBS (n = 17; control) was administered daily by oral gavages to C57BL/6 mice from 14 days before immunization with MOG_35–55_ until the end of the study. (B) R037 (0.8 mg/ml; n = 16) or PBS (n = 17) in a water bottle was administered to SJL/J mice from 14 days before immunization with PLP_131–151_ until the end of the study. Data represent mean score + SEM from a representative of two independent experiments. *p≤0.05; **p≤0.01 (t-test).

We examined the effect of the lactic acid bacterium *P. acidilactici* (R037) on the development of EAE. R037 (4 mg/day) and phosphate-buffered saline (PBS) as the control were orally administered daily to C57BL/6 mice from 14 days before immunization with mouse myelin oligodendrocyte glycoprotein (MOG_35–55_) until the end of the study. R037 treatment significantly reduced the EAE clinical score (mean peak score, 3.6±0.3 vs. 4.9±0.3; p = 0.013) (Fig. 1A; Table 1). The effect of R037 on the development of EAE was examined using another mouse strain because the effect of probiotics may differ with strains [22]. SJL/J mice were treated with 0.8 mg/ml R037 or PBS in a water bottle from 14 days before immunization with proteolipid protein (PLP_139–151_) until the end of the study. Since each mouse consumed approximately 5 ml water per day, the amount of R037 is equivalent to 4 mg/day. The suppressive effect of R037 on EAE in SJL/J mice induced with PLP_139–151_ was observed again (mean peak score, 1.5±0.2 vs. 2.9±0.4; p = 0.012) (Fig. 1B; Table 1). In addition, R037-treated SJL/J mice exhibited significantly delayed clinical onset compared to controls (13.1±0.4 vs. 11.5±0.2; p = 0.002).

**Table 1:** Clinical scores and occurrence.

	Occurrence	Onset	Peak score	AUC
C57BL/6				
R037	18/18 (100%)	13.1±0.6	3.6±0.3*	19.6±3.1*
control	17/17 (100%)	13.0±0.5	4.9±0.3	30.4±3.4
SJL/J				
R037	13/16 (81%)	13.1±0.4**	1.5±0.2*	8.4±1.4*
control	15/17 (88%)	11.5±0.2	2.9±0.4	15.2±2.2

Data are reported as mean ± SEM. The means of the peak and the area under the curve (AUC) represent data during days 1 to 21 after immunization. Data are representative of two independent experiments in C57BL/6 and SJL/J mice.

*p≤0.05;

**p≤0.01 (t-test).

**Figure 2:**
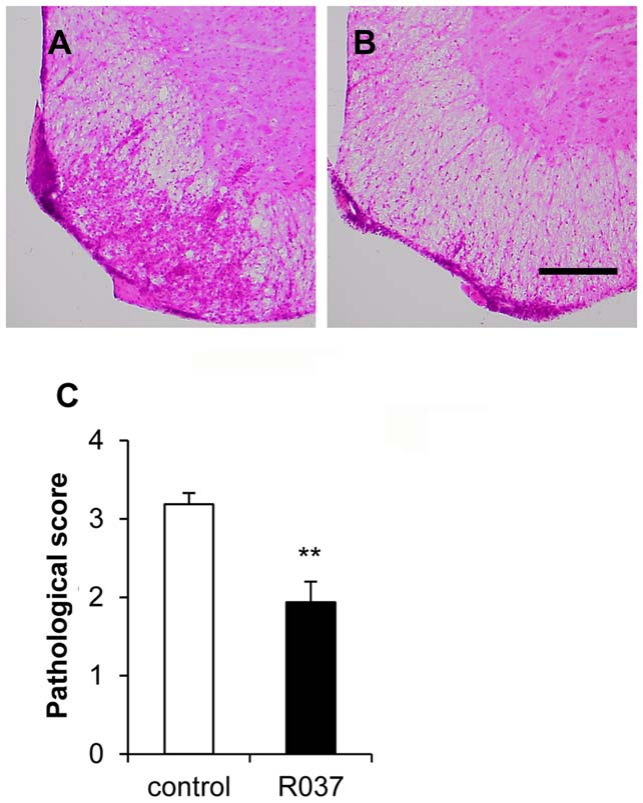
Infiltration of MNCs into the spinal cord is reduced in R037-treated mice. Spinal cord sections obtained from control (A) or R037-treated (B) C57BL/6 mice on day 22 after immunization were analyzed by hematoxyline and eosin (H&E) staining. Scale bar = 250 µm. (C) Semiquantitative evaluation of the pathological scores was performed as described in the Methods section. Each bar indicates the mean pathological score + SEM of 8 mice from each group.

Next, we examined the pathological consequences of R037 treatment. Hematoxylin and eosin (H&E) staining of the spinal cords obtained from C57BL/6 mice on day 22 after immunization showed that the number of infiltrated mononuclear cells (MNCs) in the R037 group was apparently lower than that observed in the control group (Fig. 2A, B). The significant decrease in the number of MNCs infiltrating the spinal cords of R037-treated mice was confirmed by semiquantitative analysis (1.93±0.26 vs. 3.18±0.14; p = 0.0003) (Fig. 2C). These results suggest that prophylactic treatment with R037 ameliorates EAE in at least two different mice strains.

### *P. acidilactici* inhibits the antigen-specific production of inflammatory cytokines

**Figure 3:**
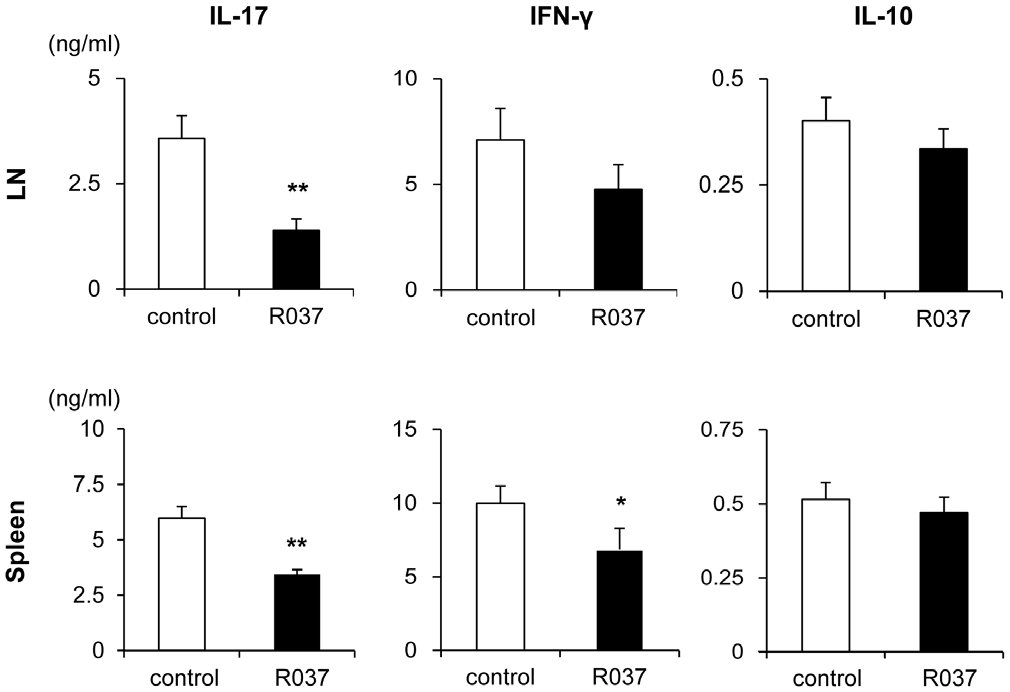
Administration of R037 inhibits the secretion of inflammatory cytokines. Lymphocytes were isolated by draining lymph nodes (dLN) and spleens of C57BL/6 mice on day 11 after immunization and restimulating with MOG_35–55_ for 72 h. IL-17, IFN-γ and IL-10 in the culture supernatants were assayed by ELISA. Decreased IL-17 production by both dLN cells and splenocytes and decreased IFN-γ by splenocytes were observed in the R037-treated group. Data are shown as mean + SEM from a representative of three independent experiments for 6 mice in the R037-treated and 8 in the control groups. **p≤0.01; *p≤0.05.

To investigate the effects of R037 on systemic inflammation, splenocytes and draining lymph node (dLN) cells harvested on day 11 after immunization were restimulated with MOG_35–55_. dLN cells obtained from R037-treated mice produced significantly less IL-17 in response to MOG_35–55_ than those obtained from the control group. The production of IFN-γ decreased in the R037-treated group although the difference was not significant. The splenocytes obtained from the R037-treated group produced significantly less IL-17 and IFN-γ than those obtained from the control group. The production of IL-10 by splenocytes and dLN cells did not differ significantly between the two groups (Fig. 3). Although we assayed IL-4 to examine the effect of R037 treatment on Th2 skewing, the levels were below the sensitivity of the assay system used for both groups (data not shown). These data suggest that R037 treatment inhibits the induction of antigen-specific encephalitogenic Th17 and Th1 cells in dLNs and spleen.

### Treatment with *P. acidilactici* induces CD4^+^ IL-10-producing cells

**Figure 4:**
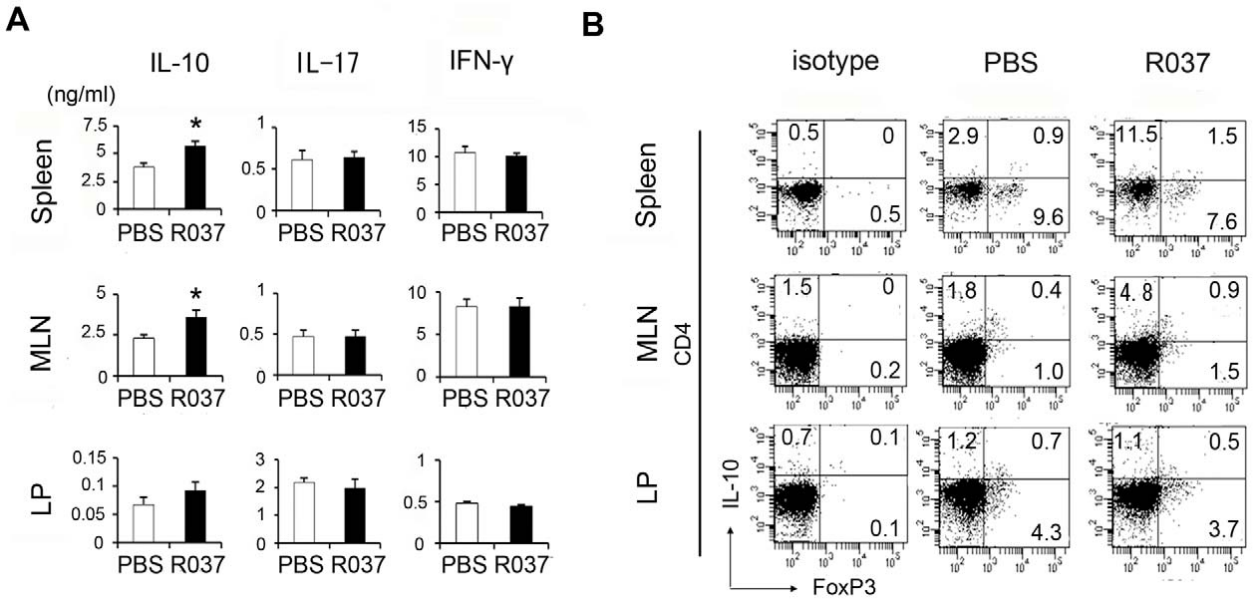
Administration of R037 induces CD4^+^ IL-10-producing T cells in mesenteric lymph nodes (MLNs) and spleen. (A) Lamina propria cells, MLNs and splenocytes were isolated from the unimmunized mice that were fed R037 for 2 weeks and after isolation, cells were stimulated with anti-CD3/anti-CD28 antibodies for 72 h in vitro. IL-17, interferon-γ (IFN-γ) and IL-10 in the supernatants were assayed by ELISA. For lamina propria, each bar indicates mean + SEM of triplicate samples from 2 mice in each group. For MLNs and splenocytes, each bar indicates mean + SEM of 8 mice in each group. Data are representative of more than three independent experiments for 8 mice each. * p≤0.05 (B) Lamina propria cells, MLNs and splenocytes were isolated from the mice that were fed R037 for 2 weeks. Intracellular staining of IL-10 and Foxp3 (B) in CD4^+^ T cells was analyzed by flow cytometry. Data are representative of 2 independent experiments.

To further elucidate how R037 suppressed systemic inflammation, we analyzed the cytokine profiles of C57BL/6 naive mice treated with R037. Lamina propria lymphocyte, mesenteric lymph node (MLN) cells and splenocytes were isolated from the mice that were fed R037 for 2 weeks and stimulated with anti-CD3/anti-CD28 antibodies for 72 h. Culture supernatants were examined; no statistical difference in the production of IL-17 and IFN-γ was observed between the R037-treated and control groups. However, a significant increase in the production of the anti-inflammatory cytokine IL-10 was observed in MLNs and splenocytes (Fig. 4A). These results suggest that R037 affects systemic immune regulation. Because R037 was administered orally, we assumed that inducible regulatory T cells (iTreg) were generated in the intestinal LP. However, no difference in the number of CD4^+^ IL-10^+^ T cells was observed between the R037-treated and control groups. On the other hand, CD4^+^ IL-10^+^ T cells apparently increased in MLNs and spleens of R037-treated mice (Fig. 4B). Next, we examined the CD4^+^Foxp3^+^ cells because Foxp3^+^ iTreg cells are generated by the use of mucosal antigens [23]. No apparent increase was observed in the number of Foxp3^+^ T cells from R037-treated mice, whereas a slight increase was observed in MLNs. These results suggest that R037 may predominantly induce Foxp3^−^ IL-10-producing cells in MLNs.

### *P. acidilactici* relieves EAE severity in a therapeutic manner

**Figure 5:**
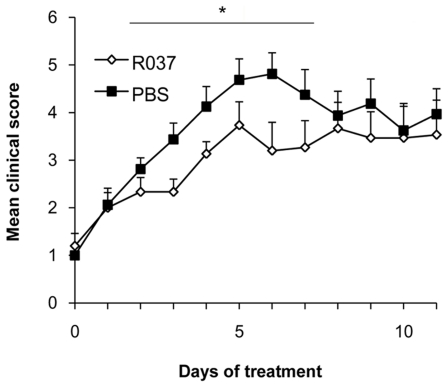
R037 relieves EAE severity in a therapeutic manner. R037 (0.8 mg/ml; n = 16) or PBS (n = 17; control) were administered in a water bottle to C57BL/6 mice from 11 days after immunization with MOG_35–55_. The area under the curve (AUC) under the bar was significantly lower in R037-treated mice. Data represent mean score + SEM of two independent experiments. *p≤0.05.

Because we have demonstrated that the prophylactic administration of R037 ameliorated EAE severity (Fig. 1), we investigated whether the bacterium exerts a beneficial effect even after clinical onset. Administration of R037 (0.8 mg/ml in a water bottle) to C57BL/6 mice with EAE was started on the day of clinical onset (day 11). Although the significant suppression assessed by mean peak scores was not observed, the R037-treated group exhibited milder clinical symptoms of EAE. Furthermore, the area under the curve (AUC) during 2 to 7 days after treatment of the R037-treated group (18.0±1.9) was significantly lower than that of the control group (24.3±2.2; p = 0.039) (Fig. 5). Thus, R037 has therapeutic and prophylactic effects on EAE.

## Discussion

In the present study, we demonstrated that the oral administration of the lactic acid bacterium *P. acidilactici* R037 induced IL-10-producing regulatory T cells and ameliorated EAE. The beneficial effect of R037 was demonstrated in C57BL/6 and SJL/J mice that were immunized with MOG and PLP peptides, respectively. In our preliminary experiments, we examined the effects of *L. delbrueckii* LAB4 and *P. acidilactici* R037 on the development of the collagen-induced arthritis model; only LAB4 exhibited a prophylactic effect (Tategaki, unpublished data). However, only R037 showed a significant suppressive effect on the EAE model in our pilot study. *L. casei* is beneficial in the collagen-induced arthritis model [18] although some controversy about its effects on EAE exists [24], [25]. In addition, the efficacy of lactic acid bacteria on EAE varied depending on the strains [19]. Taken together, these results suggest that the effects of lactic acid bacteria on a disease model appear to depend on bacterial and/or mice strains [22]. Cumulative evidence suggests a significant role of IL-17 in the pathogenesis of various autoimmune diseases, and this importance has been increasing in MS [2]. In the present study, antigen-specific production of IL-17 and IFN-γ by draining lymph nodes and splenocytes was inhibited in the R037-treated group, and the number of cells infiltrating the CNS decreased. These results suggest that orally administered R037 ameliorates EAE by suppressing systemic inflammation.

Depletion of commensal microflora by antibiotics ameliorates EAE by the induction of regulatory cells [26], [27]. Viable *P. acidilactici* could reach the intestine and produce bacteriocin, which is an antibiotic peptide [28], because they can survive in the low pH of gastric acid [29]. However, because R037 administered in our study was heat-killed, it appears to be likely that cell components of R037 increased regulatory T cells and inhibited inflammatory cytokines. The effects of bacterial components on autoimmune diseases have been reported. Polysaccharide A from *Bacteroides fragilis* is effective not only in the colitis model but also in the EAE model [30], [31]. It can induce IL-10 by CD103^+^ DCs [32]. Polymannose and mannan, which are components of the bacterial cell wall, induce IL-10 by the DCs in intestinal LP [33]. In our study, IL-10 secretion by MLNs and splenocytes derived from R037-treated mice significantly increased; moreover, an increase in the number of IL-10^+^ cells was confirmed. Increased IL-4 secretion by MLNs and splenocytes was not observed, while *L. paracasei* increased the secretion implying Th2 skewing [19]. This suggests that orally administered heat-killed R037 is effective on EAE because it induces IL-10-producing cells rather than IL-4-producing cells.

Gut-associated lymphoid tissues are thought to be the primary site for generating iTreg cells, and CD4^+^Foxp3^+^ cells were the best characterized population among these cells. Foxp3^+^ regulatory T cells play important roles by inducing anti-inflammatory IL-10-producing T cells in various autoimmune diseases [34]. Foxp3^+^ T cells induced by lactobacillus in EAE mice are thought to be critical for the beneficial effect [19]. Other regulatory CD4^+^ T cells such as T cells expressing the latency-associated protein (LAP) and Tr1 cells negative for Foxp3 have been identified [35]–[37]. CD4^+^LAP^+^ T cells are induced by the oral administration of the anti-CD3 antibody and a mixture of probiotics [16] [38]. In the present study, CD4^+^ IL-10^+^ cells were significantly increased in MLNs and spleens although CD4^+^Foxp3^+^ cells were only slightly increased in MLNs and not increased in the spleens (Fig. 4). We also stained IL-17, LAP and NK1.1 (for invariant NKT), but only a small number of cells were positively stained. And the number of IL-10-positive and one of IL-17, LAP or NK1.1-positive cells were few. Therefore, the increase of CD4^+^ IL-10^+^ cells was not due to an increase of IL-10 and IL-17 double positive cells, Th3 cells, nor invariant NKT cells. In addition, CD4^+^ IL-10^+^ cells did not increase in LP. Taken together, these results suggest that R037 may not directly induce iTreg cells in LP. Because DCs in LP migrate to MLNs by chemokines [39], it is possible that R037 induced inhibitory DCs in LP, which then migrated to MLN and primarily induced Tr-1 cells at least in C57BL/6 mice.

In addition to the prophylactic effect of R037 on EAE, R037 exhibited a beneficial effect when administered even after the clinical onset of the disease. The combination of beneficial lactic acid bacteria exhibits a synergistic effect on EAE, and viable bacteria are more effective [19]. Because the heat-killed single strain of R037 used in the present study was effective in both prophylaxis and therapy, more significant effects would be expected by using viable cells or the combination with other beneficial bacteria. Certain lactic acid bacteria including R037 have been considered edible for a long time and exist as human commensal microbiota [15]. It is important to investigate the precise mechanisms of the effects of those bacteria, and simultaneously search for more effective strains to prevent and treat multiple sclerosis.

## Materials and Methods

### Animals and Reagents

Experimental procedures were approved by the Animal Care and Use Committee of Osaka University Graduate School of Medicine (Permit Number: 20-084-4). As the C57BL6 and SJL/J mice are the typical two strains utilized in EAE experiment, female 6-week-old of both strains were obtained from Oriental Yeast Corp. (Tokyo, Japan) for this study. All mice used in this study were maintained in a specific pathogen-free environment. R037 that it is a strain of *P. acidilactici* was kindly provided by Kaneka Corp. (Osaka, Japan). Since the usage of viable bacteria is restricted in our animal facility of SPF condition, the bacteria were centrifuged, heat-killed at 80°C for 30 min before lyophilization, and stored in 4°C until use. In the R037 group, C57BL/6 mice were fed 200 µl of PBS containing 20 mg/ml of heat-killed R037 daily by single oral gavage, and SJL/J mice were fed by a water bottle containing 0.8 mg/ml heat-killed R037 from 14 days before immunization, as oral gavage administration placed great stress on SJL/J mice causing fatal consequences in some cases. In the control group, 200 µl of PBS were fed for C57BL/6 mice and only water was given in water bottle for SJL/J mice.

### EAE induction and clinical score

EAE was induced using modified method we previously described [40]. In brief, C57BL/6 mice were subcutaneously injected with 100 µg MOG (myelin oligodendrocyte glycoprotein)_35–55_ (MEVGWYRSPFSPVVHLYRNGK) peptide emulsified in complete Freund's adjuvant containing 200 µg of *Mycobacterium tuberculosis* H37Ra (Difco Laboratories, MI, USA), in addition to two intraperitoneal injections of 200 ng of pertussis toxin (List Laboratories, California, USA) on days 0 and 2. EAE in SJL/J mice was induced by subcutaneous immunization with 200 µl of a complete Freund's adjuvant containing 400 µg of H37Ra and 150 µg of PLP_139–151_ (HSLGKWLGHPDKF) distributed over three sites dorsally and on the lateral hind flanks. All mice were monitored daily for clinical signs and were scored as follows using a scale of 0–7: 0, no signs of clinical disease; 1, paralyzed tail; 2, paresis of hind limb and gait disturbance; 3, paralysis of one limb; 4, paralysis of two limbs; 5, two limbs paralyzed and paresis of a third limb, but movement still possible; 6, complete forelimb paralysis; and 7, moribund state or dead. To avoid dehydration, mice scoring more than 5 were subcutaneously administered 0.5 ml of physiological saline solution.

### Histology and semi-quantification

C57BL/6 mice were sacrificed on day 22 post immunization followed by transcardiac perfusion with 4% paraformaldehyde in PBS. Spinal cords were fixed in 4% paraformaldehyde in PBS and prepared for histological analysis. Cryosections (10-µm-thick) were stained with H&E. Semiquantitative histological analysis of inflammatory cellular infiltration was performed using the following scores: 0, no inflammation; 1, cellular infiltrates only in the perivascular areas and meninges; 2, mild cellular infiltration in parenchyma; 3, moderate cellular infiltrates in parenchyma; and 4, severe cellular infiltrates in parenchyma [41].

### Isolation of lymphocytes

Mesenteric lymph nodes, inguinal lymph nodes and splenocyte were freshly harvested and homogenized. After passed through 40 µm cell strainer, splenocyte were treated with ACK lysis buffer for 2 min. Washed with Hanks' Balanced Salt Solutions, centrifugated and pellet were used for lymphocytes. LP lymphocytes were isolated as follows [14]. Small intestines were collected and opened longitudinally, washed to remove content, and shaken in Hank's balanced salt solution containing 5 mM ethylenediaminetetraacetic acid for 20 min at 37°C. After removing epithelial cells and fat tissue, the intestines were cut into small pieces and incubated with RPMI 1640 containing 4% fetal bovine serum, 1 mg/ml collagenase D (Roche Applied Science, Indianapolis, USA), 0.5 mg/ml dispase (Invitrogen, Carlsbad, CA), and 40 µg/ml DNase I (Roche Applied Science) for 1 h at 37°C with shaking 110 rpm. The digested tissues were washed with Hank's balanced salt solution, resuspended in 5 ml of 40% Percoll (GE Healthcare, Uppsala, Sweden), and overlaid on 2.5 ml of 80% Percoll in a 15-ml Falcon tube. Percoll gradient separation was performed by centrifugation at 1800 rpm for 20 min at 25°C. The interface cells were collected and used as LP lymphocytes.

### Flow cytometry

Intracellular staining of Foxp3 and IL-10, lymphocytes were stimulated for 4 h with 50 ng/ml phorbol 12-myristate 13-acetate (Sigma, St. Louis, MO) and 1 µg/ml ionomycin (Sigma) in the presence of Brefeldin A (BD Biosciences, Franklin Lakes, NJ, USA). The cells were stained for surface CD4, fixed, and permeabilized using a BD Cytofix/Cytoperm kit, and finally stained for intracellular IL-10 and Foxp3. The following antibodies were used: APC-Cy7-labeled anti-CD4 (GK1.5, BD Biosciences), Alexa647-labeled anti-Foxp3 (FJK-16s, eBioscience), and PE-labeled anti-IL-10 (JES5-16E3, BD Biosciences) antibodies. Flow cytometry was performed using a FACS Canto-2 ™ with Diva ™ software (Becton Dickinson).

### Cytokine assay

For the assessment of antigen-specific cytokine production, MNCs were isolated from draining LNs and spleens of C57BL/6 mice on day 11 after immunization with MOG_35–55_. Cells were restimulated with the peptide for 72 h, and IL-17, IFN-γ, IL-4, and IL-10 were assayed by enzyme-linked immunosorbent assay (ELISA, R&D Systems for IL-17, IFN-γ, and IL-4, eBioscience for IL-10). For the assay of cytokines produced by lymphocytes obtained from R037-treated naive mice, MNCs from lamina propria, MLNs and spleens were isolated from mice that were fed R037 for 2 weeks and stimulated with coated 1 µg/ml anti-CD3 and soluble 1 µg/ml anti-CD28 antibodies for 72 h. Next, IL-17, IFN-γ, IL-4 and IL-10 were assayed by ELISA. All assays were performed in triplicate.

### Statistical analysis

Data in Figure 1A, B and Figure 5, the area under the curve (AUC) were calculated. For statistical analysis, Data in Figure 2C, 3 and Figure 5, student's t-test was used to calculate significant levels. Data in Figure 4A, one way ANOVA was used to calculate significant levels. p≤0.05 was considered statistically significant.
